# The use of complementary and alternative medicine by patients with cancer: a cross-sectional survey in Saudi Arabia

**DOI:** 10.1186/s12906-018-2150-8

**Published:** 2018-03-12

**Authors:** Khadega A. Abuelgasim, Yousef Alsharhan, Tariq Alenzi, Abdulaziz Alhazzani, Yosra Z. Ali, Abdul Rahman Jazieh

**Affiliations:** 1 King Abdullah International Medical Research Center, Riyadh, Saudi Arabia; 2 Department of Oncology, Ministry of National Guards, King Abdulaziz Medical City, Riyadh, Saudi Arabia; 3 King Saud bin Abdulaziz University for Health Sciences, Riyadh, Saudi Arabia

**Keywords:** Complementary and alternative medicine, Religious belief, Cancer, Camel products, Brucellosis, Middle East respiratory syndrome coronavirus (MERS-CoV)

## Abstract

**Background:**

A significant proportion of cancer patients use complementary and alternative medicine (CAM) along with conventional therapies (CT), whereas a smaller proportion delay or defer CT in favor of CAM. Previous studies exploring CAM use among cancer patients in the Middle East region have shown discrepant results. This study investigates the prevalence and pattern of CAM use by Saudi cancer patients. It also discusses the possible benefits and harm related to CAM use by cancer patients, and it explores the beliefs patients hold and their transparency with health care providers regarding their CAM use.

**Methods:**

A cross-sectional study was conducted in oncology wards and outpatient clinics by using face-to-face interviews with the participants.

**Results:**

A total of 156 patients with a median age of 50 years (18–84) participated in the study. The prevalence of CAM use was 69.9%; the most prominent types of CAM were those of a religious nature, such as supplication (95.4%), Quran recitation (88.1%), consuming Zamzam water (84.4%), and water upon which the Quran has been read (63.3%). Drinking camel milk was reported by 24.1% of CAM users, whereas camel urine was consumed by 15.7%. A variety of reasons were given for CAM use: 75% reported that they were using CAM to treat cancer, enhance mood (18.3%),control pain (11.9%), enhance the immune system (11%),increase physical fitness (6.4%), and improve appetite (4.6%). Thirty percent of CAM users had discussed the issue with their doctors; only 7.7% had done so with their nurses.

**Conclusions:**

The use of CAM, including camel products, is highly prevalent among cancer patients in the Middle East, but these patients do not necessarily divulge their CAM use to their treating physicians and nurses. Although CAM use can be beneficial, some can be very harmful, especially for cancer patients. Association is known between camel products and brucellosis and Middle East respiratory syndrome coronavirus (MERS-CoV). Both can lead to tremendous morbidity in immune-compromised patients. Doctor–patient communication regarding CAM use is of paramount importance in cancer care.

**Electronic supplementary material:**

The online version of this article (10.1186/s12906-018-2150-8) contains supplementary material, which is available to authorized users.

## Background

The National Center for Complementary and Integrative Health (NCCIH) defines complementary medicine as a non-mainstream practice used together with conventional medicine and defines alternative medicine as a non-mainstream practice used in place of conventional medicine [1, 2]. The use of CAM is widespread worldwide, ranging from 9.8% to 76%, and the pattern of use differs according to socioeconomic status, geography, and religious and spiritual beliefs [3, 4]. Among cancer patients, 10% to 80% reported CAM use, with prayer and nutritional supplements being the most common [5–9].

Patients with cancer may prefer CAM, which they might perceive as being “safe and natural” to chemotherapy and radiation therapy, which can alarm some patients. These patients do not necessarily understand that “natural” does not always mean “safe”. Moreover, alternative practitioners can be perceived as caring, with a holistic interest in the patient’s well-being, whereas oncologists tend to direct their discussions with their patients toward specific aspects of the disease [10, 11].

CAM use is widely practiced the in the Saudi community (33–93.3%) [12–17]. The majority of CAM users in the Saudi community obtain their information about CAM from friends, families, and the media [10]. The majority of CAM used in Saudi Arabia (dietary and non-dietary) are of a religious nature, such as supplication and recitation of the Quran, consumption of Zamzam water (water from holy Mecca), regularly drinking water upon which the Quran has been read, and cupping/hijama. Drinking honey, camel milk, and camel urine, which are all mentioned in the Holy Quran as therapeutic measures, are also commonly used in Saudi Arabia [10]. The Ministry of Health (MOH) provides free medical services for all Saudis, excluding traditional healers’ and CAM costs. CAM practice is regulated by the National Complementary and Alternative Medicine Center established by the MOH [18].

Only two previous studies have explored CAM use among cancer patients in Saudi Arabia, and both showed very discrepant results [16, 17]. It is unclear whether the differences in reported prevalence and CAM types are due to interregional differences or related to differences in the surveys used.

There has been a recent noticeable increase in the publicity of camel products in Saudi Arabia due to the common belief in the positive effects of them in different diseases. Camel products have been linked to brucellosis and Middle East respiratory syndrome coronavirus (MERS-CoV); however, Saudis continue to consume camel products. During the 2014 MERS-CoV outbreak, all cancer patients who encountered the infection in King Abdulaziz Medical City, Riyadh, died from the infection (a total of 15). We have also noticed increasing numbers of brucellosis infections in our cancer patients. In the previous two papers, the issue of camel products (milk and urine) was not fully addressed [16, 17]

The aim of this study was to reevaluate CAM use among Saudi patients with cancer and to explore the patients’ insights and beliefs about CAM use. We also sought to assess the willingness of patients with cancer to divulge their use of CAM to their nurses and physicians.

## Methods

### Ethical approval

Approval for this study was provided by the King Abdullah International Medical Research Center Institutional Review Board (RC 16/165/R). Written informed consent was given by all participants prior to participation in the study.

### Setting

This cross-sectional, survey-based study was conducted in two oncology wards, with 24 beds in each, one used for patients with solid tumor and the other for patients with hematological malignancies and stem-cell transplantation, and in oncology outpatient clinics at the King Abdulaziz Medical City in Riyadh.

### Participant selection

Adult patients with confirmed cancer diagnoses, specifically hematological malignancies and solid tumors, were approached in person between July 1 and December 31, 2016, and were asked about their willingness to participate after explaining the purpose of the study. Patients who were actively receiving and those who had completed cancer-directed therapy within the past year were asked to participate. Patients who were known to have mental disorders were excluded.

### Tool

A previously developed questionnaire used by Jazieh AR et al. (2012), which was based in Jazieh AR et al. (2004), was modified to include patients with hematological malignancies and those undergoing hematopoietic stem-cell transplantation (HSCT), translated into Arabic and translated back to English for consistency [17, 19]. The questionnaire was administered to 15 participants and was subsequently adjusted to overcome inconsistency and to ensure reliability.

The research coordinators collected demographic and disease profile data from medical records and used the validated questionnaire in a face-to-face interview with the participants (Additional file 1).

During the interview, participants were asked about specific practices and therapies they use for their cancer, other than what is prescribed by their treating doctors, including Quran recitation; supplication; or consumption of any dietary or non-dietary materials or supplements, including Zamzam water, water upon which the Quran has been read, black seeds (*Nigella sativa),* camel milk, camel urine, garlic, olive oil, multivitamins, known herbal remedies, and unknown herbal mixtures.

We allowed participants to mention any form of CAM not listed in the questionnaire and listed those as “others.” The questionnaire also included items exploring participants’ perception and beliefs about CAM and transparency with health care providers regarding CAM use.

### Statistical analysis

Responses to all questions were reviewed to avoid missing data before the end of each participant’s interview. Data were entered into a Microsoft Access database and peer reviewed for quality checking. Validity constraints were applied to the database to ensure accuracy and consistency. The data were then exported to IBM SPSS Statistics for Windows, version 22.0 (Armonk, NY, USA) for analysis.

Frequencies of nominal categorical variables were compared by the Chi-square test, and continuous variables were compared by *t*-tests. Inferential analysis was performed by applying univariate analysis to examine the association between CAM use and demographic and clinical characteristics. Predictors of CAM use were also investigated by multivariate logistic regression. *P* values < 0.05 were considered statistically significant.

## Results

### Participant characteristics

A total of 156 cancer patients participated in the study. About half of the participants were females (85; 54.4%) with a median age of 50 years (range 18–84). Compared to our institution’s tumor registry, the gender distribution of our participants was almost equal, whereas the median age was slightly different (57 years). The demographic and clinical characteristics are summarized in Table 1.

**Table 1:** Participants’ characteristics (*n* = 156)

Characteristic		Number	Percent
Gender	Male	71	45.5
	Female	85	54.5
Marital status	Married	123	78.8
	Single	33	21.1
Education level	Uneducated	37	23.7
	Higher education	47	30.1
	Lower education	72	46.1
Work status	Not working	82	52.6
	Working	38	24.4
	Retired	36	23.1
Monthly income	≤SR 6000	59	37.8
	>SR 6000	93	59.6
	Rather not state	4	2.6
Smoking	Current	17	10.9
	Former	23	14.7
	Never smoked	116	74.4
Cancer type	Solid tumor	128	82.1
	Hematological malignancy	28	17.9
Cancer stage	Non-metastatic	94	60.3
	Metastatic	34	21.8
	NA	28	17.9
Type of treatment	Surgery	90	57.7
	Radiation	69	44.2
	Chemotherapy	131	84
	Stem cell transplant	16	10

Higher education: college and postgraduate, lower education: elementary to high school, NA: not applicable, SR: Saudi riyals

### Pattern of CAM use

The prevalence of the use of CAM was 69.9%, with a confidence interval of 62.02% to 76.95%.The majority of CAM users (71%) started using CAM after being diagnosed with cancer. Only 3.7% of participants indicated that they had delayed cancer treatment in favor of CAM use. The most common reported types of CAM were those of a religious nature, such as supplication, Quran recitation, Zamzam water, and water upon which Quran has been read. Among non-religious CAM, olive oil was the most commonly used, followed by black seeds (*Nigella sativa*) (Table 2).

**Table 2:** Frequency of use of different types of complementary and alternative medicine (CAM)

		Number	Percent
CAM use	Users	109	69.9
	Non-users	47	30.1
Types of CAM	Supplication	103	94.5
	Quran recitation	96	88.1
	Zamzam water	92	84.4
	Water read upon Quran	69	63.3
	Olive oil	61	56.0
	Black seed (*Nigella sativa*)	40	37.0
	Garlic	36	33.3
	Camel milk	26	24.1
	Honey	24	22.0
	Camel urine	17	15.7
	Known herbal remedies	16	14.7
	Multivitamins	11	10.1
	Unknown herbal mixture	4	3.7
	Others^a^	6	5.5

^a^Dates, turmeric, mushrooms, and yogurt

**Fig. 1:**
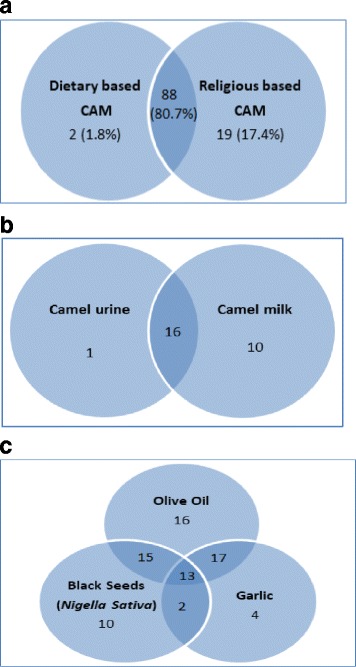
Venn Diagrams demonstarting overlapping usage of different types of complementary and alternative medicine (CAM). Dietary vs.religious based CAM use (**a**); camel product users tend to use both (**b**); dietary based CAM overlap (**c**)

There was a clear overlap between different types of CAM used. The majority of participants used dietary and religious-based CAM concurrently, whereas only 1.8% used only dietary CAM. Almost all camel urine users (94.1%) also used camel milk; garlic users commonly used it with olive oil, with black seeds (*Nigella sativa*), or with both (Fig. 1).

A univariate analysis was conducted to investigate the effects of demographic and clinical factors on CAM use. We excluded Quran recitation and supplication from this univariate and multivariate association analysis, because their use was very common (supplication 95.4%; Quran recitation 88.1%). CAM was significantly associated with gender (62.2% of CAM users were female, compared with 37.8% male, *p* = 0.011), employment status (57.1%of CAM users were unemployed, 26.5% employed, and 16.3% retired, *p* = 0.034), and radiation therapy (54.1% of CAM users had received radiotherapy, 45.9% had not, *p* = 0.008) (Table 3).

**Table 3:** Univariate analysis of the association between complementary and alternative medicine (CAM) use and demographic and clinical factors

		CAM use				
		No		Yes		*p*
		*n*	%	*n*	%	
Age (years) median (range)		55 (18–84)		49 (19–83)		0.051
Gender	Male	34	58.6	37	37.8	0.011^a^
	Female	24	41.4	61	62.2	
Marital status	Married	44	75.9	79	80.6	0.483
	Unmarried	14	24.1	19	19.4	
Education level	Higher education	15	25.9	32	32.7	0.650
	Lower education	29	50.0	43	43.9	
	Uneducated	14	24.1	23	23.5	
Work status	Not working	26	44.8	56	57.1	0.034^a^
	Working	12	20.7	26	26.5	
	Retired	20	34.5	16	16.3	
Monthly income	≤SR 6000	21	37.5	38	39.6	0.799
	>SR 6000	35	62.5	58	60.4	
Cancer type	Solid tumor	46	79.3	82	83.7	0.493
	Hematological malignancy	12	20.7	16	16.3	
Solid tumor stage	Non-metastatic	36	78.3	58	70.7	0.355
	Metastatic	10	21.7	24	29.3	
Surgery	No	23	39.7	43	43.9	0.606
	Yes	35	60.3	55	56.1	
Radiation	No	42	72.4	45	45.9	0.001^a^
	Yes	16	27.6	53	54.1	
Chemotherapy	No	12	20.7	13	13.3	0.222
	Yes	46	79.3	85	86.7	

SR: Saudi riyals

^a^The Chi-square statistic is significant at < 0.05

Higher education: college and postgraduate, Lower education: elementary to high school

Multivariate logistic regression analysis confirmed a relationship with CAM use for both gender and radiation therapy (OR = 3.375, *p* = 0.029; and OR = 3.036, *p* = 0.015, respectively). In addition, the analysis showed that younger age and not having received surgery were also predictors of CAM use (Table 4).

**Table 4:** Multivariate logistic regression analysis of between complementary and alternative medicine (CAM) use and demographic and clinical factors

				95% C.I. for OR	
		*p*	OR	Lower	Upper
Age		0.032*	0.960	0.926	0.996
Gender	Female vs. male	0.029*	3.375	1.129	10.087
Marital status	Married vs. unmarried	0.175	2.119	0.716	6.269
Education	Higher education vs. uneducated	0.856	1.143	0.271	4.818
	Lower education vs. uneducated	0.448	0.634	0.196	2.055
Work status	Unemployed vs. employed	0.583	1.380	0.438	4.347
Monthly income	≤SR 6000 vs. >SR 6000	0.242	1.822	0.667	4.981
Cancer stage	Non-metastatic vs. metastatic	0.979	0.987	0.359	2.710
Surgery	Yes vs. no	0.030*	0.318	0.113	0.896
Radiation	Yes vs. no	0.015*	3.036	1.237	7.451
Smoking status	Current or former vs. never smoked	0.254	2.133	0.580	7.853

SR: Saudi riyals

*The *p* value is significant at< 0.05

### Participants’ beliefs regarding CAM use

Among cancer patients who used CAM, a variety of reasons were given for this decision: 75% reported that they were using CAM as a treatment for cancer. Other reported reasons were to improve mood (18.3%), to control pain (11.9%), to improve the immune system (11%), to increase fitness (6.4%), and to improve appetite (4.6%). Although 28 (25.7%) reported no improvement after CAM use, the remaining 81 (74.3%) reported some overlapping improvement in terms of enhancement of mood (56.8%), appetite (23.5%), physical strength (7.4%), and immunity (2.5%) as well as reduction of pain (33.3%). Among those who felt some improvement with CAM use, 77.8% attributed the improvement to both cancer treatment and CAM use, whereas 14.8% believed their improvement was related to medical therapy and only 7.4% thought that CAM use was the only reason for their perceived improvement.

Among the patients who did not use CAM, 40.4% stated that CAM use did not cross their minds when they were considering treatment options, 31.9% were advised by their doctor not to use CAM, 29.8% did not think that CAM use was beneficial, 4.3% were not aware of CAM use for cancer, and only 2.1% mentioned cost as the main reason for not using CAM.

### Divulgence of CAM use to healthcare workers

Whereas 19% of CAM users reported that they had discussed the issue with their doctors, only 8% and 3% had done so with their educator or nurse, respectively. Among those who discussed their CAM use with their doctors, 42.9% perceived their doctor’s response to be supportive, 33.3% unsupportive, and 23.8% neutral. Patient educator’s responses were perceived as unsupportive in 44.4% of the cases, supportive in 33.3%, and neutral in 22.2%.The nurses’ responses were perceived to be equally distributed among supportive, unsupportive, and neutral.

## Discussion

Interest in CAM is growing worldwide, especially in the era of the Internet and social networking, which is rich in reports by patients of their positive personal experiences of CAM [20, 21]. Despite tremendous progress in cancer therapy [20], a significant proportion of patients with cancer use CAM along with their medical therapies [9, 21]. Although 71% of CAM users started their CAM use after cancer diagnosis, the remaining 29% reported starting CAM use prior to their cancer diagnosis, which concurs with the previously reported high rate of CAM use in the Saudi general population [12].

Previous studies exploring CAM use among Saudi cancer patients are limited and of small sample sizes. However, they showed variable results; Sait et al. showed a prevalence of only 20%, whereas Jazieh et al. suggested that CAM use is highly prevalent (reaching 90%) [16, 17]. In our study, the prevalence of CAM use was 69.9%, and the most prominent types of CAM were those of a religious nature. The great degree of variability between the two studies is similar to the variability in CAM use reported by different studies in the Saudi community and could be explained by the studies’ distinct methodologies and the fact that the majority of those studies were conducted in single institutions [12].

CAM has some beneficial outcomes in cancer patients, such as pain relief, nausea control, mood enhancement, and control of fatigue [22–24]. However, there are potential risks when cancer patients use some types of CAM, especially when they are actively receiving conventional therapies for their cancer. Some dietary herbs can lead to severe hepatotoxicity necessitating liver transplantation and even death [25, 26]. Liver damage related to hepatotoxic herbs can be further augmented with some conventional cancer therapies and vice versa, and some herbal remedies, such as gingko, kava, ginseng, and garlic, may interfere with liver metabolism of anticancer drugs, leading to either decreasing cancer drug therapeutic effect or augmenting toxicities [27].

Brucellosis is very common in the Middle East region, and it has been directly linked to contact with camel urine and consumption of unheated camel milk [28–33]. The fatal Middle East respiratory syndrome coronavirus (MERS-CoV) has been linked to contact with camels and consumption of raw camel milk [34, 35]. Although we did not ask in this study whether the milk was heated prior to consumption, it is a known custom in Saudi Arabia to drink camel milk raw. Among CAM users in this study, 94.1% of those who drink camel urine also use camel milk. In the Middle East region, it is paramount for health care workers, especially those caring for cancer patients, to discuss with their patients the potential risks of using camel products.

Previous studies exploring CAM use among Saudi cancer patients were conducted prior to the outbreak of MERS-Co. Sait et al. did not explore other important CAM such as camel products, whereas Jazieh et al. reported an 8.6% rate of camel urine use and 9.3% milk use without mentioning the milk source [16, 17].

Evidence suggests that a considerable number of CAM users do not discuss this practice with their healthcare providers [16]. In our study, only one-third of CAM users divulged their CAM use to their treating physicians, and fewer patients informed their educators and nurses. Healthcare workers might not discuss CAM use with their patients because they are either busy or are not well-informed about CAM. In addition, patients with cancer and their families likely expect that most healthcare workers, including oncologists, do not support the use of CAM despite its widespread acceptance in the general community. It is important for CAM use to be routinely explored with all patients with cancer who are undergoing active conventional therapy, given the compromised immune status of such patients, the possible interaction with conventional cancer therapy, and the potential CAM-related liver toxicity.

Although some health care workers might be completely against CAM, it is evident that CAM use is beneficial in many aspects of cancer patients’ lives [24] .In this study, three-quarters of our CAM users (74.3%) felt some improvement in different aspects such as mood (56.8%), appetite (23.5%), physical strength (7.4%), and immunity (2.5%) as well as reduction of pain after CAM use. Among those who felt some improvement, 7.4% thought that CAM use was the only reason for their perceived improvement, whereas 77.8% attributed the improvement to both conventional cancer therapies and CAM use, and 14.8% believed their improvement was related to conventional cancer therapy.

Only small numbers of patients with cancer adopt CAM treatments in lieu of mainstream therapy [12]. In our study, only 3.7% of CAM users delayed conventional cancer-directed therapy. However, we interviewed patients who attended oncology clinics or who were admitted to the oncology floor, and these patients were likely to have received conventional therapies for their cancers. A large, community-based study should provide a better estimate of the number of patients who adopt CAM instead of conventional therapy.

Our study has some limitations: first, the small sample size in a single institution may not be representative of the Saudi community; second, recruiting patients attending a cancer center will miss cancer patients who elect to use CAM only without seeking medical advice.

## Conclusions

The use of CAM is highly prevalent among cancer patients in the Middle East and includes camel products, but these patients do not necessarily divulge their CAM use to their treating physicians and nurses. Although CAM use can be beneficial, some can be very harmful, especially to cancer patients. Association between camel products and brucellosis and MERS-CoV is well-known. Both can lead to tremendous morbidity in immune-compromised patients; therefore, doctor–patient communication regarding CAM use is paramount in cancer care.

## Additional file

Additional file 1: A previously developed questionnaire used by Jazieh AR et al. (2012), which was based in Jazieh AR et al. (2004), was modified to include patients with hematological malignancies and those undergoing hematopoietic stem-cell transplantation (HSCT), translated into Arabic and translated back to English for consistency. (DOCX 42 kb)

## Abbreviations

CAM: Complementary and alternative medicine
CT: Conventional therapies
HSCT: Hematopoietic stem-cell transplantation
MERS-CoV: Middle East respiratory syndrome coronavirus
