# Supplementary material for: The use of complementary and alternative medicine by patients with cancer: a cross-sectional survey in Saudi Arabia
Source: BMC Complement Altern Med. 2018 Mar 12;18:88. doi: 10.1186/s12906-018-2150-8 (PMC5848536; doi:10.1186/s12906-018-2150-8)
Supplement: Supplementary file 1 — A previously developed questionnaire used by Jazieh AR et al. (2012), which was based in Jazieh AR et al. (2004), was modified to include patients with hematological malignancies and those undergoing hematopoietic stem-cell transplantation (HSCT), translated into Arabic and translated back to English for consistency. (DOCX 42 kb) [file 12906_2018_2150_MOESM1_ESM.docx]

D**ate of Survey ---/----/----- Name of Surveyor_____________________**

1. **القسم الأول First Section**

| **Subject’s serial number #: _______________** | | | |
| --- | --- | --- | --- |
| **تاريخ الميلاد Date of Birth** | **----- / ------/ ------ (اليوم/ الشهر/ السنة) (day/ month/ year)** |  |  |
| **الجنس Gender** | **ذكر Male  أنثى Female** |  |  |
| **الحالة الاجتماعية Marital Status** | **أعزب/عزباء Single  متزوج/ة Married  مطلق/ة**  **Divorced**  **أرمل/ة Widow  منفصل/ة Separated** |  |  |
| **المستوى التعليمي Education Level** | **غير متعلم/ة Uneducated إبتدائي Primary  متوسط Intermediate  ثانوي Secondary  تعليم عالي Higher Education** |  |  |
| **حالة العمل Work Status** | **بوظيفة Job With  بلا وظيفة With no Job** |  |  |
| **الدخل الشهري Monthly Income** | **˂ 3000  3000 -6000  6000 > أُفضل عدم الذكر Rather not state** |  |  |
| **هل أنت مدخن/ة؟ Smoking** | **نعم Yes  توقفت Stopped  لم أدخن من قبل Never smoked** |  |  |
| **نوع السرطان Type of Cancer** | **سرطان في الدمBlood cancer  سرطان صلب Solid tumor غير ذلك :Otherـــــــــــــــــــــــــــــــــــــــــــــــــــــــــــــــــــــــــــــــــــــــــــــــــــــــــــــــ** |  |  |
| **مرحلة السرطان Stage of Cancer** | **منتشر Metastatic  غير منتشر Non- Metastatic غير منطبق Not applicable** |  |  |
| **ما نوع العلاج الذي تلقيته؟ What type of treatment did you receive?** | **عملية جراحية: Surgery  نعم Yes  لا No** |  |  |
|  | **علاج بالإشعاع: Radiation  نعم Yes  لا No** |  |  |
|  | **علاج بالكيماوي Chemotherapy  نعم Yes  لا No** |  |  |
|  | **زراعة خلايا جذعية: Stem Cell Transplant  نعم Yes  لا No** |  |  |

| 1. **ا 2- القسم الثانيSecond section** | |
| --- | --- |
| **العلاج التكميلي/ البديل هو أي ممارسة مثل الصلاة, الدعاء, الإبر الصينية, التدليك و ما إلى ذلك, أو أي منتج مثل الفيتامينات, الأملاح المعدنية, الأعشاب, الأحماض الأمينية, المنتجات النباتية و ما إلى ذلك ,**  **Complementary and alternative therapy is any practice, such as prayer, supplication, Chinese needles, massage and so on, or any product such as vitamins, mineral salts, herbs, amino acids, plant products and so on** | ***.*** |
| - **هل تتناول أي علاجات تكميلية أو بديلة؟ Do you use any complementary or alternative therapy?**   **لا, حدد السبب : □ لا أعلم عنه شيئا □ لم أفكر بالأمر □ لأنه مكلف □ لا أظن أنه مجد □ أخبرني الطبيب أنه غير نافع □ أسباب أخرى------------**  **NO, why? I don’t know about it  It didn’t cross my mind  It is costly  I on’t think it is good My doctor told me it is not good Other reasons, specify--------**  **YES , please answer the below questions:**  **نعم , الرجاء الإجابة على الاسئلة التالية وتحديد الأنواع المستخدمة:** | |
| - **هل بدأت بالاستخدام قبل المرض؟ نعم لا**   **بعدالمرض؟ نعملا**  **Have you started using before illness?  Yes No**  **After Illness  Yes No** |  |
| - **هل أجلت العلاج الطبي حتى تستخدم العلاج التكميلي/ البديل أو لا**؟  **نعملا**   **Did you delay your treatment in order to use complementary and alternative treatment?  Yes No** |  |
| - **كم التكلفة التقريبية الشهرية للعلاج التكميلي/ البديل***:------------* ***ريالا***   **What is the approximate monthly cost for complementary and alternative treatment: ----------- SR** |  |
| - **هل ناقشت تناولك إياها مع الطبيب المعالج**؟ ** نعم**  ** لا** - **كيف كانت رده فعله؟ مساند غير مساند محايد**   **Did you discuss complementary and alternative therapy with your doctor? No  Yes**  **What was his reaction? supports doesn’t support Neutral** |  |
| - **هل ناقشت تناولك إياها مع الممرضة؟  نعم لا** - **كيف كانت رده فعله؟ مساند غير مساند محايد**   **Did you discuss using complementary and alternative therapy with your nurse?  Yes**  **No**  **What was his reaction?  supports doesn’t support Neutral** |  |
| - **هل ناقشت تناولك إياها مع المثقفة الصحية ؟  نعم  لا** - **كيف كانت رده فعلها ؟ مساندة غير مساندة محايدة**   **Did you discuss complementary and alternative therapy with your patient educator? No  Yes**  **What was his reaction? supports doesn’t support Neutral** |  |

1. **القسم الثالث Third section**

| **من أين عرفت عن العلاجات التكميلية أو البديلة؟**  ***(يمكن اختيار اكثر من اختيار)***  **How did you find out about it? (check all that apply)** | **عادات ومعتقدات اجتماعية  عادات ومعتقدات دينية  ممارس طب شعبيأحد أفراد العائلة / صديق  التلفاز / الانترنت  رجل دين أخرى , الرجاء التحديد:--------------------------------------------**  **Social Believes Religious Believes Herbal Medicine Practitioner Family member TV/ Internet Religious Scholar Other, specify------** |
| --- | --- |
| **أنواع علاج الطب التكميلي والبديل:**  ***(يمكن اختيار اكثر من اختيار)***  **Types of Complementary Alternative Medicine**  **(check all that apply)** | **الدعاء الرقية الشرعية ماء زمزم ماء مقروء عليه قرانالحبة السوداءحليب الابلبول الابلثومزيت زيتونمكملات غذائيةأعشاب معروفةخلطات أعشاب جاهزة غير معروفة المصدر أخرى , الرجاء التحديد:----------------------------------------------------------**  **Supplication Quran recitation  Zamzam water  Water read upon Quran**  **Black seed Camel milk Camel urine Garlic Olive oil Multivitamin**  **Known herbal remedies Unknown herbal mixture Other, specify------------** |
| **أسباب استخدام العلاج التكميلي أو البديلي ؟**  ***(يمكن اختيار اكثر من اختيار)***  **Reasons for Complementary Alternative Medicine use (check all that apply)** | **تقليل حجم الورم تخفيف شدة الألم  علاج المرض زيادة الشهية  زيادة القوة الجسدية**  **زيادة المناعة معتقدات دينية  تحسين المزاج  معتقدات اجتماعية  أخرى , الرجاء التحديد:---------------------------------------------------------------**  **Decrease tumor size Decrease pain Treat cancer Increase appetite Increase physical strength  Increase immunity Religious believes Improve mood level Social believes** |
| **نوع التحسن الذي شعرت به بعد استخدام العلاج التكميلي والبديل**  ***(يمكن اختيار اكثر من اختيار)***  **How do you feel an improvement after using Alternative and Complementary treatment? (check all that apply)** | **خف الألم تحسنت الشهية**  **تحسنت الحالة النفسية  زيادة قوة العظام  لم ألاحظ أي تحسن**  **أخرى , الرجاء التحديد:---------------------------------------------------------------**  **Decreased pain  Enhanced appetite  Enhanced mood Enhanced physical strength I did not see any benefit Other, specify---------** |
| **ما هو في اعتقادك سبب هذا التحسن:**  ***(يمكن اختيار اكثر من اختيار)***  ***What do you think the reason for improvement?***  **(check all that apply)** | **العلاج التكميلي اوالبديل الذى استخدمته العلاج الطبي الذى وصفه طبيبك المعالج  كليهما معا**  **Complementary and alternative treatment used**  **Medical treatment given by your treating doctor Both of them** |

**Completed By ________________________________**
